# Impact of the Cross-Curricular Education Program on Food Intake, Physical Activity, and Body Mass Index of School Children in a Local Community in Northern Serbia

**DOI:** 10.3390/children8110947

**Published:** 2021-10-21

**Authors:** Sanja Šumonja, Marija Jevtić

**Affiliations:** 1 College for Vocational Studies in Subotica, 24000 Subotica, Serbia; 2 Faculty of Medicine, University of Novi Sad, 21000 Novi Sad, Serbia; marija.jevtic@uns.ac.rs; 3 Institute of Public Health of Vojvodina, 21000 Novi Sad, Serbia; 4 Research Center on Environmental and Occupational Health, School of Public Health, Université Libre de Bruxelles (ULB), 1050 Brussels, Belgium

**Keywords:** assessment, education, nutrition, school-aged population, sedentary time

## Abstract

Introduction: The trends in the state of nutrition and physical activity of school-age children in Serbia impose a need to create and evaluate programs to improve their nutrition and physical activity. The purpose of this study was to evaluate the impact of the cross-curricular nutrition and physical activity education program (NPAEP) on food intake, physical activity, and body mass index in first–fourth-grade primary school children. Material and methods: The study used an experimental pre-test (t1) post-test (t2) design. The sample included 167 participants in t1 and 178 in t2. The differences in food intake, physical activity, and body mass index before and after implementation of the cross-curricular nutrition and physical activity education program were analyzed using the Mann–Whitney U test, the Chi-squared test, and the Wilcoxon signed-rank test. Values *p* < 0.05 were considered statistically significant. Results: Fruit (t1 = 0.70 ± 0.55; t2 = 1.26 ± 0.65; *p* < 0.001) and dairy intake (t1 = 1.52; t2 = 1.79; *p* = 0.005) significantly increased in the intervention group. A significant reduction in time spent watching television (t1 = 78.0; t2 = 56.7; *p* = 0.005) and playing on the computer (t1 = 32.3; t2 = 25.8; *p* = 0.047) was achieved in the intervention group. Time spent in organized sports activities significantly increased only in the intervention group (t1 = 21.9; t2 = 30.5; *p* = 0.046). Body mass index did not change significantly in the intervention group after the implementation of the NPAEP. Conclusions: The nutrition and physical activity education program contributed to an increase in fruit and dairy intake and time spent in intense physical activities and a reduction in screen time. The presented results justify the application of the NPAEP in improving the nutrition and physical activity of first–fourth-grade primary school children.

## 1. Introduction

Improper diet and lack of physical activity are some of the most important health risk behaviors of school-aged children in Serbia as well as in other parts of Europe and the world. According to the NCD Risk Factor Collaboration, there is a noticeable increase in prevalence of overweight and obesity among children and adolescents in Serbia [1]. A national study conducted in 2013 on a sample of 7–14-year-old children showed that 14.5% of children and adolescents were overweight and 13.7% of them were obese [2]. A more recent study of health-risk behavior in school-aged children in Serbia showed that 18.5% of adolescents are overweight and 5.7% are obese [3]. Furthermore, the same study revealed that less than half of adolescents consume fruit and vegetables at least once a day [3]. Children and adolescents in Serbia spend a significant amount of time in screen-based sedentary activities [3]. The current trends in nutritional status and physical activity of school children in Serbia indicate a need for creating and evaluating programs to improve nutrition and physical activity of school-aged children.

There have been no implemented or evaluated population-based measures or programs to improve nutrition and physical activity of school-aged children in Serbia so far. Programs for improving nutrition and physical activity in schools in Serbia are usually carried out through short-term programs that are not evaluated [4]. The lack of properly addressed content about nutrition and physical activity in the primary school curricula in Serbia is partly due to the workload of teachers and pupils in other school subjects and content. 

The largest number of factors that positively affect the habits related to nutrition and physical activity of children can be included in a program implemented in schools, because schools are educational institutions where, besides home, children spend most of their time. Therefore, schools are the most common place where nutrition and physical activity improvement programs are implemented [5]. Previously published studies conclude that the most effective programs for improving nutrition and physical activity of school-aged children are those that combine learning across the curriculum and have an impact on parents or school environment [6]. Integrated education of children and parents through a cross-curricular approach may be a solution for implementing nutrition and physical activity education in elementary schools. A cross-curricular approach promotes cognitive, emotional, and social development, and is particularly interesting for teaching content outside the regular curriculum [7]. The main advantage of a cross-curricular approach in teaching is that it does not require more classes and time children have to spend at school. If teachers are well prepared, a cross-curricular approach does not put an additional burden on their engagement in work [7,8]. 

The purpose of this study was to evaluate the impact of the cross-curricular nutrition and physical activity education program on food intake, physical activity, and body mass index of first–fourth-grade school children in Serbia. This research attempts to answer whether the nutrition and physical activity education program based on a cross-curricular approach:

– may significantly increase fruit, vegetable, and dairy intake of primary school children;
– may significantly increase the time primary school children spend in moderate and intense physical activities;
– may significantly reduce screen-based sedentary time of primary school children;
– may significantly reduce the number of overweight and obese primary school children.

The research is based on the following hypothesis:

**Hypothesis** **1** **(H1).** *The NPAEP can significantly increase intake of fruit and vegetables in school-aged children*.

**Hypothesis** **2** **(H2).** *The NPAEP can significantly increase intake of dairy in school-aged children*.

**Hypothesis** **3** **(H3).** *The NPAEP can significantly increase time spent in moderate and intense physical activity in school-aged children*.

**Hypothesis** **4** **(H4).** *The NPAEP can significantly reduce screen-based sedentary time in school-aged children*.

**Hypothesis** **5** **(H5).** *The NPAEP can significantly reduce the number of overweight and obese school-aged children.*

## 2. Material and Methods

### 2.1. Participants

The participants were recruited from primary schools in a local community in Northern Serbia. All five primary schools from the local community were invited to participate in the study and two schools accepted. The defined target population included all first–fourth-grade pupils from both schools (*N* = 736). The study sample was formed using the two-stage cluster sampling method [9]. Each class in every grade was one cluster, which means that the population consisted of 32 clusters (16 classes, e.g., clusters). Each class was delivered a number and put in a box. There were four boxes, one for each grade. Two classes from each grade were selected to participate in the study by pulling classes number out of the box. The teachers and parents from the selected classes were informed about the study protocol on a meeting with the researchers. All the parents and teachers were provided an informational letter containing a written consent form. All the pupils whose parents returned the informed written consent were eligible for inclusion in the study. After obtaining permissions from the parents and pupils, the total sample included 8 classes with 167 children in t1 (before the implementation of the NPAEP) and 178 in t2 (after the implementation of the program).

The participants completed pre-test assessments. Following pre-test assessments (t1), the clusters from each grade were randomly assigned to be intervention (the I group) or the control group (the C group). The participants from the I and C groups were not significantly different according to gender, age, and body mass index. 

### 2.2. Study Design 

The study was designed as an experimental study pre-test (t1—time one) post-test (t2—time two) with two groups (intervention—the I group, and control—the C group). The research was conducted during the 2014–2015 school year. This study was approved by the Ethics Committee of the Faculty of Medicine in Novi Sad (at the 57th session of the Committee for the Ethics of Clinical Research on 4 December 2014) and the local School management (reference number: 424-614-0450/2014-15).

### 2.3. Intervention

The intervention in this study was a Nutrition and Physical Activity Education Program (NPAEP) created for first–fourth-grade primary school children and their parents. The NPAEP was designed as a cross-curricular program. The idea of the cross-curricular program is based on a cross-curricular teaching approach, which means that the knowledge and skills about nutrition and physical activity are developed through different subject areas simultaneously. The current guidelines for conducting nutrition education in schools were used as a theoretical framework for creating the Nutrition and Physical Activity Education Program [10,11]. The Food and Agriculture Organization (FAO) recommends that school-based nutrition education should include various educational strategies which encourage active participation, students’ practical activity, experience exchange and learning outside the classroom [11]. 

The development of the NPAEP went through two phases. In the first phase, the researchers analyzed the current curricula for first–fourth grades of primary school in Serbia in order to find content that could be used to integrate messages about nutrition or physical activity. The second phase included defining aims, content, teaching methods, recommended evaluation methods, and duration for each module and class. The NPAEP included 45 to 50 lessons in each grade organized in six modules: the roles of food, food groups, principles of proper nutrition, planning nutrition, safety food handling, and physical activity.

The part of the NPAEP for parents included 4 to 5 workshops. The workshops included activities and discussion on the consequences of inadequate nutrition and physical inactivity on children’s health and academic achievement, influence of different parental styles, and home environment on children’s eating habits, principles of proper nutrition, tips for preparing healthy meals, etc. There were three workshops organized only for parents at parent teacher meetings, and two workshops organized with the children and parents together.

Three independent researchers with expertise in nutrition and education evaluated the NPAEP for content validity. Their suggestions were incorporated before the final version of the NPAEP was created. 

The NPAEP was implemented during one school year (nine months) in the I group classes. The C group classes worked as usual. The NPAEP was carried out by the teachers and researcher together. The researchers prepared the teachers to deliver content in several sessions. After the implementation of the NPAEP, the participants from the I and C group completed the post-test assessments (t2). 

### 2.4. Outcomes

The outcome measures were fruit, vegetable, and dairy intake; physical activity; and body mass index of first–fourth-grade children.

Fruit, vegetable, and dairy intake, as well as screen-based sedentary and physical activities, were analyzed using the questionnaire “My food and activities for one day” that was developed previously. The questionnaire was a combination of 24-h food recall and food and activity recognition form. The validity of the questionnaire was analyzed elsewhere [12,13]. Body mass index (BMI) was calculated based on anthropometric measurements obtained during physical education class. The researchers measured children’s weight and height using weighing scale with altimeter model SECA SE-711. Following the WHO recommendations, the children were categorized according to their BMI as underweight, normal weight, overweight, and obese [14,15,16].

### 2.5. Statistical Analysis

The statistical analysis was conducted using SPSS version 13. Food intake was expressed as a mean intake of servings of fruit, vegetable, and dairy for three days. Physical activity was represented as a mean number of minutes spent in screen-based sedentary activities (watching TV, playing computer games, mobile phone, or other electronic devices), spontaneous playing outside (moderate physical activities), and organized sports activities (intense physical activities). The differences in average intake of fruit, vegetable and dairy, as well as time spent in screen-based sedentary and physical activities between the I group and the C group before and after the implementation of the NPAEP, were analyzed using the Mann–Whitney U test and a Chi-squared test (weight status). The Wilcoxon signed-rank test was used to analyze changes in fruit, vegetable, and dairy intake, as well as time spent in screen-based sedentary and physical activities in the I group and the C group from t1 to t2. The differences in BMI between the I group and the C group from t1 to t2 was analyzed using the Chi-squared test. The Chi-squared test was also used to analyze differences in fruit, vegetable, and dairy intake; screen-based sedentary and physical activities; and BMI between boys and girls and different grade groups (the first–second-grade and the third–fourth-grade group). Values *p* < 0.05 were considered statistically significant.

## 3. Results

The total sample included 167 participants in t1 and 178 participants in t2. Sample characteristics in t1 are presented in Table 1. There were no significant differences found in distribution of the participants from the I group and the C group according to gender and age in t2.

### 3.1. Fruit, Vegetables and Dairy Intake

Table 2 shows average intake of fruit in the I group and the C group in t1 and t2. The intake of fruit increased significantly in the I group in t2 (*p* < 0.001).

Average daily intake of vegetables is presented in Table 3. The differences in average intake of vegetables in first–second grade and third–fourth grade subgroups of the I group and the C group in t1 and t2 are presented in Table 3.

**Figure 1:**
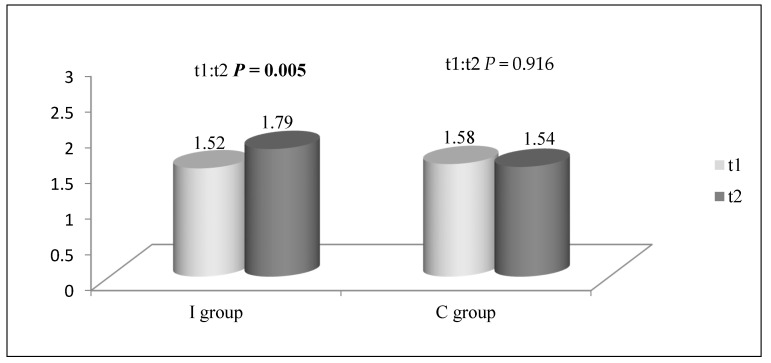
Average daily intake of dairy (servings) in the I group and the C group in t1 and t2.

Figure 1 represents average daily intake of dairy in the I group and the C group in t1 and t2. The dairy intake was significantly higher in the I group than the C group in t2 (*p* = 0.022). The participants from first and second grades from the I group reported significantly higher intake of dairy in t2 than in t1 (t1 = 1.72; t2 = 2.00; *p* = 0.029). The intake of dairy did not change significantly in third and fourth grades of the I group in t2 (t1 = 1.29; t2 = 1.56; *p* = 0.054).

### 3.2. Screen-Based Sedentary and Physical Activities

**Figure 2:**
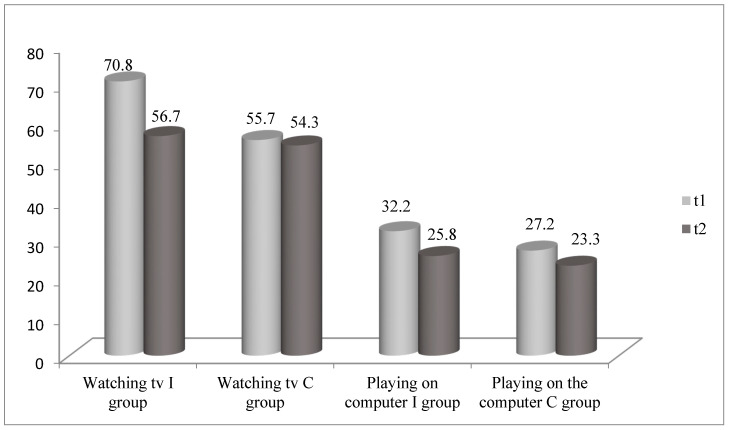
Average time spent in screen-based sedentary activities in the I group and the C group in t1 and t2.

The average time spent in screen-based sedentary activities in the I group and the C group in t1 and t2 is presented in Figure 2. The participants from the first and second grade of the I group spent significantly less time watching TV (t1 = 71.2 min; t2 = 60.2 min; *p* = 0.046) and playing on the computer or their phone (t1 = 27.4 min; t2 = 20.3 min; *p* = 0.027) in t2 than in t1. The girls from the I group reported significantly less time spent watching TV in t2 than in t1 (t1 = 72.4 min; t2 = 50.8 min; *p* = 0.002).

Table 4 shows average time (min.) spent playing outside (moderate physical activities) in the I group and the C group in t1 and t2.

**Figure 3:**
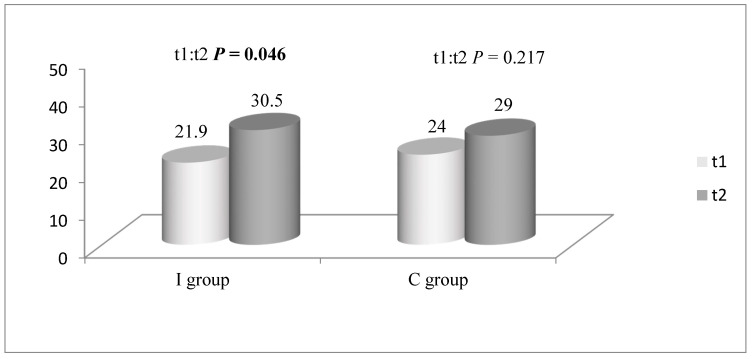
Average time (min.) spent in organized sports activities in the I group and the C group in t1 and t2.

The average time (min.) spent in organized sports activities (intense physical activities) in the I group and the C group is presented in Figure 3. The third and fourth grade participants from the I group reported significantly more time spent in organized sports activities in t2 than in t1 (t1 = 19.4 min; t2 = 37.3 min; *p* = 0.004).

### 3.3. Body Mass Index

**Figure 4:**
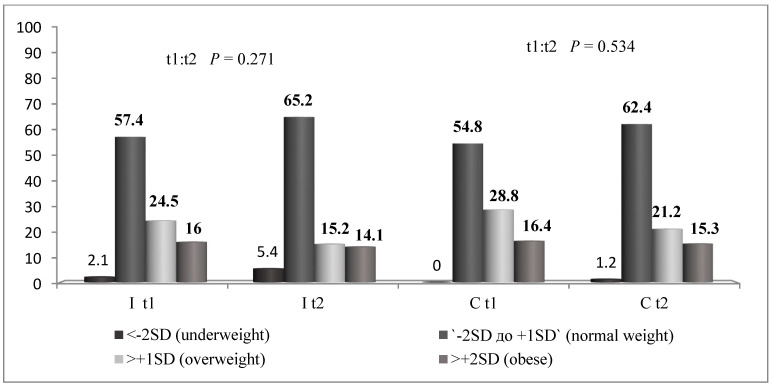
Weight status of the participants from the I group and the C group in t1 and t2.

Distribution of BMI in the I group and the C group is presented in Figure 1. The Chi-square test did not show significant changes in BMI from t1 to t2 in both groups (Figure 4).

There were no statistically significant differences found in BMI of the boys and girls from the I group (boys *p* = 0.18; girls *p* = 0.79) and the C group (boys *p* = 0.77; girls *p* = 0.49).

The BMI was not significantly different in any grade subgroup of the I group and the C group in t1 and t2.

## 4. Discussion

The average daily intake of fruit was below recommendations in both groups before the NPAEP, although fruit intake was significantly higher in the C group than in the I group. After the implementation of the NPAEP (t2), the fruit intake significantly increased in the I group and stayed unvaried in the C group. Children are more prone to consuming fruit than vegetables, which may be one of the reasons why increasing fruit consumption is easier to achieve in intervention studies [1,4,17]. An extensive international study showed that younger children consume more fruit than older children in relation to daily recommendations [18]. Before the NPAEP, fruit intake was approximately one serving a day in the first–second-grade participants from the I group and the C groups. After the implementation of the NPAEP, the first–second-grade children from the I group significantly increased fruit intake (for around half of a serving a day), which was higher than the fruit intake of their peers from the C group. Before the NPAEP, the third–fourth-grade children from the C group consumed significantly more fruit daily than the third–fourth graders from the I group. After the NPAEP implementation, the fruit intake of the third–fourth-grade children from the I group and the C group was not significantly different because the fruit intake of the third–fourth-grade participants from the I group increased considerably. These results confirm that younger children are more prone to consuming fruit and that the NPAEP had a significant impact on the fruit intake of first–fourth-grade children [4,17,18].

The vegetable intake was far below recommendations in the I group and the C group before the NPAEP, which confirmed earlier findings that insufficient vegetable intake in school-age children is a cause for concern [18,19,20,21]. The intake of vegetables in the C group was significantly higher than the I group before the NPAEP. After the implementation of the NPAEP, the vegetable intake increased in both groups although the increase was statistically significant only in the I group. Different factors, such as seasonal variations in availability of fresh vegetables or tendency for pleasing research goals, may have had an influence on reporting a higher vegetable intake after the implementation of the NPAEP. The significantly higher intake of vegetables in the I group may also be a consequence of the implementation of the NPAEP. 

By observing the impact of the NPAEP on vegetable intake in relation to grade groups, this study revealed that the NPAEP influenced vegetable intake of children from all grades. Vegetable intake was not significantly different among the participants from the I group and the C groups of first–second and third–fourth grades before the NPAEP. After the implementation of the NPAEP, the vegetable intake significantly increased among the first–second-grade children from the I group and the C group, although the increase was higher in the I group. The intake of vegetables of the first–second-grade participants from the I group was quite close to the recommended intake of vegetables for the given age, and was significantly higher than the vegetable intake of their peers from the C group after the NPAEP. Although the vegetable intake significantly increased among the third–fourth-grade participants from the I group, it was not statistically higher than the vegetable intake of the third–fourth-grade participants from the C group.

Acknowledging that the previously mentioned factors may have influenced the increase in vegetable intake, these results indicate that the NPAEP had an impact on vegetable intake in first–fourth-grade children. A higher impact on vegetable intake of younger children is expected, given that younger children generally consume more vegetables probably due to a greater parental influence [20].

The dairy intake did not reach recommendations in both groups before the NPAEP. These results are in line with the findings of the national study conducted by the Institute for Public Health of Serbia, which show that low intake of dairy is one of the problems of school children’s diet in Serbia [2,22]. Longitudinal studies show a trend of decreasing dairy intake in school-aged children [15,23]. After the NPAEP, the dairy intake significantly increased in the I group and remained unchanged in the C group, indicating that the NPAEP may have influenced the intake of dairy. By analyzing the dairy intake of different grade groups, it was noticed that the daily intake of dairy was not significantly different in the first–second- and third–fourth-grade participants from the I group and the C group. However, the dairy intake was significantly higher only in the first–second grade participants from the I group probably due to the implementation of the NPAEP. These results may partly be explained by the fact that dairy intake decreases with age or may indicate that the NPAEP was more suitable for first and second grade children [23]. By analyzing results by gender, this study also showed that dairy intake significantly increased in the boys but not in the girls from the I group. This may be expected considering that boys are more likely to consume dairy than girls [24].

Taking current recommendations for physical activity for school-aged children into account, the participants from the I group and the C group did not exceed the upper limit for time spent in sedentary activities in t1 and t2. However, the participants from the I group spent significantly more time in screen-based sedentary activities than their peers from the C group before the NPAEP. It was difficult to compare these results with other similar results from Serbia, given there are not enough data on physical activity and sedentary behavior of a representative sample of school-aged children in Serbia. A national study conducted by the Institute for Public Health of Serbia analyzed the percentage of children who participated in organized sports activities or regular physical education classes, but did not include an analysis of time spent in sedentary activities [2]. Another study conducted by the same institution on a representative national sample of children from fifth and seventh grade and first year of secondary school revealed that nearly 40% of adolescents spent more than two hours a day in screen-based sedentary activities [3]. Other studies on physical and sedentary activities of school-aged children in Serbia used smaller samples, different age groups, and a different methodology for collecting information [25,26]. A study conducted on a sample of overweight and obese adolescents showed that they spent nearly 5 h a day in sedentary activities [26]. Nevertheless, studies from other countries confirm that sedentary behavior of school-aged children is important to health risk behavior related to an increased risk of cardiovascular diseases, cancer, obesity, and overall mortality rate [27]. 

After the implementation of the NPAEP, the time spent in screen-based sedentary activities significantly decreased in the I group. By analyzing the results according to grade groups, it was noticed that the reduction in screen-based sedentary time was statistically significant in the first–second grade I group, but not in the third–fourth grade I group. These results indicate that the implementation of the NPAEP could lead to a reduction in screen-based sedentary time in younger children. There remains a question why the NPAEP did not have any influence on screen-based sedentary time in the third–fourth-grade children, whether the lack of influence was due to the duration of the program, its content, or something else. Review studies show that, in general, school-based programs to improve physical activity have a positive effect on reducing time spent in sedentary activities in 6–12-year-old children [28]. It has been known that screen-based sedentary behavior becomes a more serious problem as the children grow [18]. It is possible that it requires a different strategy and methods to influence screen-based sedentary behavior of older children. 

The implementation of the NPAEP significantly reduced screen-based sedentary time in girls but not in boys. Different studies show that, while girls generally spend more time in screen-based sedentary activities, boys are more prone to screen-based sedentary activities, such as playing games on the computer or on phones [18]. It is important to find an appropriate approach to influence boys’ screen-based sedentary time.

Before the NPAEP, the participants from the I group spent 89 min on average in moderate physical activities, which was significantly more than 46 min that was the average time spent in moderate physical activities for the C group participants. These differences may be a reflection of variations in time that the children spend in moderate physical activity or errors in parental estimation of the time children spent playing outside. Working hours or tendency towards giving socially desirable answers may be some of the factors that influenced the accuracy of parents’ estimation of time their children spend physically active [12,29]. After the NPAEP, the time spent in moderate physical activities significantly increased in both the I group and the C group. Since a significant increase in time spent in moderate physical activities was registered in both groups, it may be supposed that factors other than the NPAEP influenced it. It may be possible that weather conditions were more favorable in t2 (May) and, therefore, that children spent more time playing outside. School obligations may have decreased more in t2 than in t1 (September), so children had more free time to spend playing outside. By analyzing time spent in moderate physical activities according to the grade groups (Table 4), it is noticeable that differences in time children spent in moderate physical activities were significant only between the first–second-grade participants from the I group and the C group. 

The time spent in organized sports activities, e.g., intense physical activities, significantly increased in the I group after the NPAEP. Since the time spent in intense physical activities did not change significantly in the C group, it may be assumed that the implementation of the NPAEP influenced the time children spend in organized sports activities. It may be possible that the parental awareness about the importance of physical activities in childhood increased after the NPAEP, so more children started playing sports. The time spent in organized sports activities increased considerably in the third–fourth-grade participants from the I group and remained unchanged in the first–second-grade group. These results indicate that the introduction of the NPAEP in the third–fourth-grade group could lead to an increase in time spent in intense physical activities. There remains a question why the NPAEP was not as influential in increasing time spent in intense physical activities in younger children. Review studies show that school-based programs to improve physical activity may increase the time children spent in moderate or intense physical activities [17,28]. 

Figure 4 shows that almost a half of the participants from this study were not in the category of normal weight, confirming the importance of an increasing trend of childhood overweight and obesity in Serbia [2]. After the implementation of the NPAEP, there were no statistically significant changes found in the weight status of the participants from the I group and the C group. In both groups of respondents, a slight trend of decreasing number of obese children and increasing number of normal weight children was observed. These results indicate that changes in BMI are probably due to factors other than the NPAEP, since they were observed in both the I group and the C group. The changes in BMI were not observed in gender or grade subgroups of the sample. The duration of the implementation or the design of the NPAEP may be some of possible reasons why the NPAEP did not have a significant impact on BMI. A systematic review by Silveira and colleagues showed that the studies that can lead to significant changes in weight status of school children have some common characteristics: last longer than one year, include nutrition curriculum in regular school curriculum and other activities, include collaboration with parents, and increase availability of fruit and vegetables in school meals [30]. The NPAEP included nutrition curriculum in the regular school curriculum and other school activities, and was based largely on collaboration with parents. The results of this study indicate that the parents’ awareness of importance of proper nutrition and physical activity in childhood was not sufficient to bring long-term changes in child nutrition and physical activity, and, consequently, their BMI. The ability of parents to provide proper nutrition and opportunities for physical activity for their children is largely a reflection of their socio-economic status. Therefore, it is important that primarily schools and local community offer their contribution by increasing availability of fruit and vegetables in schools and opportunities for physical activity. 

### 4.1. Limitations

It is important to keep in mind some limitations and advantages when interpreting results of this study. The sample included children from one local community and, therefore, results may not be representative for the population of school children in Serbia. Since the program was delivered during one school year, it is not expected to have a long-term influence on children’s nutrition and physical activity. 

The accuracy of the food intake and physical activity reported by parents and children was tested in previous studies [12,13]. The results and suggestions from those studies were used to create the final version of “My food and activity for one day”. The data on child nutrition and physical activity were collected over three consecutive days. The fact that they were participating in the research may have had an impact on child nutrition and physical activity for the data collection days. 

An important advantage of this study is its design involving two parallel groups: the intervention and the control group. This design allowed us to compare changes in food intake and physical activity during one school year between the group that received the NPAEP (I group) and the group that worked according to the usual curriculum. 

### 4.2. Conclusions 

The NPAEP based on a cross-curricular approach contributed to a significant increase in intake of fruit, vegetables, and dairy in the intervention group.

The time spent in screen-based sedentary activities decreased considerably in the intervention group after the NPAEP. The decrease in the time spent in screen-based sedentary activities was more significant in the girls and the participants from the first–second-grade subgroup of the intervention group.

The time spent in moderate and intense physical activities was significantly higher in the intervention group after the NPAEP. The increase in the time spent in intense physical activities was more significant in the third–fourth-grade subgroup of the intervention group.

The number of overweight and obese children did not change significantly after the NPAEP in the intervention group.

### 4.3. Recommendations for Practice

The results of this study showed that the NPAEP may be a useful and effective method to improve nutrition and physical activity of school-aged children. 

The NPAEP can be incorporated into the regular school curriculum without increasing the time children spent at school or overall school workload. 

Implementing the program on a broader sample of school-aged children in Serbia during a continuous first cycle of elementary education is recommended in order to evaluate the long-term impact on health risk behaviors related to inadequate diet and physical activity and to determine sustainability of the program.

## Figures and Tables

**Table 1:** Sample distribution according to gender and grade in t1.

t1	First Grade *p* = 0.355			Second Grade *p* = 0.483		
Gender	I	C	Total	I	C	Total
Boys	52% (13)	41.2% (7)	47.6% (20)	50% (12)	44.4% (8)	47.6% (20)
Girls	48% (12)	58.8% (10)	52.4% (22)	50% (12)	55.6% (10)	52.4% (22)
Total	100% (25)	100% (17)	100% (42)	100% (24)	100% (18)	100% (42)
**t1**	**Third Grade *p* = 0.208**			**Fourth Grade *p* = 0.619**		
Gender	I	C	Total	I	C	Total
Boys	57.1% (12)	38.9% (7)	48.7% (19)	50% (12)	50% (10)	50% (22)
Girls	42.9% (9)	61.1% (11)	51.3% (20)	50% (12)	50% (10)	50% (22)
Total	100% (21)	100% (18)	100% (39)	100% (24)	100% (20)	100% (44)

**Table 2:** Average intake of fruit (servings) in the I group and the C group in t1 and t2.

Time	Group	First–Second Grade	Third–Fourth Grade	Total Sample
t1	I	0.88 ± 0.49 (0.0–2.3)	0.51 ± 0.54 (0.0–2.0)	0.70 ± 0.55 (0.0–2.3)
	C	1.05 ± 0.63 (0.0–2.5)	1.29 ± 0.77 (0.0–3.0)	1.18 ± 0.71 (0.0–3.0)
*p*	I:C	0.142	<0.001	<0.001
t2	I	1.39 ± 0.67 (0.0–3.0)	1.11 ± 0.59 (0.0–2.5)	1.26 ± 0.65 (0.0–3.0)
	C	1.04 ± 0.56 (0.0–2.0)	1.07 ± 0.50 (0.0–2.5)	1.05 ± 0.53 (0.0–2.5)
*p*	I:C	0.011	0.653	0.028
*p(I)*	t1: t2	<0.001	<0.001	<0.001
*p(C)*	t1: t2	0.955	0.277	0.440

**Table 3:** Average daily intake of vegetables (servings) in first–second-grade and third–fourth-grade subgroups of the I group and the C group in t1 and t2.

Time	Group	First–Second Grades	Third–Fourth Grades	Total Sample
t1	I	0.67 ± 0.35 (0.1–1.7)	0.77 ± 0.66 (0.0–4.0)	0.72 ± 0.52 (0.0–4.0)
	C	0.71 ± 0.30 (0.0–1.3)	0.95 ± 0.39 (0.0–1.5)	0.83 ± 0.37 (0.0–1.5)
*p*	I:C	0.473	0.012	0.011
t2	I	1.24 ± 0.55 (0.2–2.7)	1.06 ± 0.54 (0.0–3.0)	1.15 ± 0.55 (0.0–3.0)
	C	0.95 ± 0.35 (0.2–1.8)	1.05 ± 0.40 (0.2–2.0)	1.00 ± 0.38 (0.2–2.0)
*p*	I:C	0.006	0.758	0.074
*p(I)*	t1:t2	<0.001	0.003	<0.001
*p(C)*	t1:t2	0.003	0.492	0.016

**Table 4:** Average time (min.) spent playing outside (moderate physical activities) in the I group and the C group in t1 and t2.

Time	Group	First–Second Grade	Third–Fourth Grade	Total Sample
t1	I	107 ± 74.9 (0.0–300)	68.9 ± 63.6 (0.0–270)	89.0 ± 72 (0–300)
	C	32.9 ± 38.1 (0.0–160)	57.9 ± 48.7 (0.0–240)	45.9 ± 45.4 (0–240)
*p*	I:C	<0.001	0.567	<0.001
t2	I	183 ± 77.2 (40–420)	121 ± 76.6 (0–260)	154 ± 82.6 (0–420)
	C	109 ± 64.6 (0–240)	97.8 ± 59.6 (0–240)	104 ± 62.0 (0–240)
*p*	I:C	<0.001	0.170	<0.001
*p(I)*	t1:t2	<0.001	0.001	<0.001
*p(C)*	t1:t2	<0.001	0.001	<0.001
